# Lifestyle Factors, Genetic Risk, and Cardiovascular Disease Risk among Breast Cancer Survivors: A Prospective Cohort Study in UK Biobank

**DOI:** 10.3390/nu15040864

**Published:** 2023-02-08

**Authors:** Hexiang Peng, Siyue Wang, Mengying Wang, Xueheng Wang, Huangda Guo, Jie Huang, Tao Wu

**Affiliations:** 1 Department of Epidemiology and Biostatistics, School of Public Health, Peking University, Beijing 100191, China; 2 School of Public Health and Emergency Management, Southern University of Science and Technology, Shenzhen 518055, China

**Keywords:** lifestyle factors, cardiovascular disease, genetic risk, breast cancer

## Abstract

Background: Evidence is limited regarding the association between lifestyles and cardiovascular disease (CVD), and the extent to which healthy lifestyles could offset the genetic risk of CVD in females with breast cancer (BC). Methods: Females diagnosed as BC, who were free of CVD at baseline, from UK Biobank were included. Five modifiable lifestyle factors were considered to calculate the healthy lifestyle score, namely body mass index (BMI), smoking, alcohol drinking, dietary habits, and physical activity. The polygenetic risk score (PRS) was derived for coronary heart disease (CHD), ischemic stroke (IS), and heart failure (HF). Results: In 13,348 female BC survivors, there were 986 CVD events (736 CHD, 165 IS, and 353 HF) over a median of 8.01 years of follow-up. Participants with 4–5 healthy lifestyle components were associated with a decreased risk of incident CVD (HR: 0.50; 95%CI: 0.37, 0.66), CHD (HR: 0.49; 95%CI: 0.35, 0.69), IS (HR: 0.35; 95%CI: 0.19, 0.65), and HF (HR: 0.59; 95%CI: 0.36, 0.97), compared with those with 0–1 lifestyle components. Evidence for the genetic–lifestyle interaction was observed for CHD (*p* = 0.034) and HF (*p* = 0.044). Among participants at high genetic risk, a healthy lifestyle was associated with a lower risk of CHD (HR: 0.37; 95%CI: 0.24, 0.56), IS (HR: 0.37; 95%CI: 0.15, 0.93) and HF (HR: 0.39; 95%CI: 0.21, 0.73). Conclusions: Our findings suggest that BC survivors with a high genetic risk could benefit more from adherence to a healthy lifestyle in reducing CVD risk.

## 1. Introduction

The incidence of breast cancer (BC) in women has increased by approximately 0.5% per year, and it has surpassed lung cancer as the most diagnosed cancer, accounting for one third of all female cancer cases [1,2]. Early diagnosis and intensive treatment are reported to improve the prognosis and prolong the survival of BC patients, and the 5-year relative survival rate of female BC is as high as 90% [1] and the 20-year survival rate is 89% with standard treatment [3]. It is not enough to focus solely on cancer progression in BC patients over such a long survival period. There are many factors influencing the short- and long-term survival of BC patients, and adherence to a healthy lifestyle might improve their survival time and quality [4]. Moreover, the increased risk for cardiovascular diseases (CVDs) and CVD-related mortality in BC survivors has been widely reported [5,6,7]. BC patients diagnosed with CVD have a 60% higher mortality rate than BC patients without CVD [8]. In addition, the onset of CVD could accelerate the progression and metastasis of BC [8]. The risk of CVD deaths is even higher than that of BC deaths among BC survivors [9]. Therefore, it is imperative to remind BC patients of the risk of subsequent CVD and encourage preventive measures such as following a healthy lifestyle, which might improve their adherence to an overall healthy lifestyle and promote the survival of BC patients.

Both lifestyle factors and genetics jointly affect the occurrence and development of CVD [10,11]. Previous studies demonstrated that adherence to healthy lifestyle factors could effectively reduce the risk of CVD [12,13]. Traditional modifiable healthy lifestyle factors, including avoiding overweight or obesity, no smoking, moderate alcohol consumption, regular physical activity, and keeping a healthy dietary pattern, are shown to be the best strategies to prevent the risk of CVD [14,15,16,17,18]. Additionally, previous genome-wide association studies suggested that genetic factors play an essential role in the pathogenesis of CVD, and the polygenic risk score (PRS) might accurately predict the risk of CVD [19,20,21,22]. Furthermore, the genetic risk of CVD might be attenuated by a healthy lifestyle, suggesting that advocating for a healthy lifestyle could offset the genetic risk and benefit the primary prevention of diseases [23]. BC patients might benefit from a healthy lifestyle to prevent the subsequent CVD risk and offset the genetic risk of CVD. Therefore, it is necessary to provide evidence of whether a healthy lifestyle could attenuate the genetic risk of CVD among BC patients.

However, the role of adhering to a healthy lifestyle in CVD prevention and to what extent a healthy lifestyle can offset CVD genetic risk in BC patients is less explored in prospective settings [24]. We hypothesized that females with BC could also benefit from a healthy lifestyle, which might improve their adherence to an overall healthy lifestyle and prevent the subsequent burden of cardiovascular complications. Therefore, we aimed to investigate the association between healthy lifestyle factors and CVD risk among female BC survivors from a large population-based cohort study (UK Biobank). In addition, we further explored whether healthy lifestyle patterns might modify the association between the PRS of CVD and the risk of incident CVD.

## 2. Methods

### 2.1. Participants and Study Design

UK Biobank is one of the largest cohort studies in the world, with the details of the study protocol reported previously [25]. The baseline survey of UKB was conducted from 2006 to 2010 across the United Kingdom, with over 0.5 million participants aged 40–69 enrolled in the cohort. The participants were recruited from 22 assessment centers in England, Wales, and Scotland, where they participated in the baseline survey. All the participants completed their baseline questionnaires, underwent various anthropometric measurements, and reported medical conditions. The online website (www.ukbiobank.ac.uk, accessed on 20 December 2022) summarizes the information about the cohort and collected variables. The demographic information, lifestyle factors, and other health-related covariates were collected using a standardized electronic questionnaire.

The current analyses included all females with BC at baseline. BC cases were identified by self-reported information, the operation information, and the International Classification of Diseases (174 and 2330 for ICD9, and C50 for ICD10). In the present study, subjects with coronary heart disease (CHD), heart failure (HF), stroke, angina, myocardial infarction, cerebrovascular disease, or other cancer at baseline were excluded.

The North West Multicenter Research Ethical Committee approved the UK Biobank study. This study was conducted according to the Declaration of Helsinki. Generic ethical approval was obtained by UK Biobank from the NHS National Research Ethics Service (approval letter dated 17 June 2011, Ref 11/NW/0382). All participants provided written informed consent to participate in UK Biobank. We applied for the related data according to the rules of the UKB data sharing policy, and the data sharing agreement with the UKB is represented by the approval code 66,137.

### 2.2. Assessment of Lifestyle Factors and Other Covariates

Five traditional modifiable lifestyle factors were considered in the current study, namely body mass index (BMI), smoking, alcohol drinking, dietary habits, and physical activity. A standardized touchscreen questionnaire was used to assess the lifestyle factors at baseline. For BMI, the high-risk group was defined with BMI ≥ 25 kg/m^2^. Those with no current smoking were considered a low-risk group. For alcohol drinking, those with a daily alcohol consumption < 14 g were defined as non-excessive alcohol drinkers, which were considered low-risk. For dietary habits, we generated a dietary score using the following five dietary habits: consumption of vegetables ≥ 4 servings/day, fruits ≥ 3 servings/day, fish ≥ 2 servings/week, processed meat ≤ 2 servings/week, unprocessed red meat ≤ 1 serving/week. A dietary score ≥ 4 was considered to reflect a healthy diet; otherwise, it was defined as an unhealthy diet. The low-risk group for physical activity included those engaged in at least 150 min of moderate physical activity or at least 75 min of vigorous physical activity per week. The lifestyle definition above has been used in previously published studies [26,27].

A healthy lifestyle score was defined according to the number of low-risk lifestyle factors, ranging from 0 to 5, with a higher score indicating a healthier lifestyle. The healthy lifestyle score was subsequently divided into four groups (0–1 points, 2 points, 3 points, and 4–5 points).

Information for covariates was obtained using qualified questionnaires [28], including sociodemographic characteristics (age, sex, race, education, income, menopause), medical history (hormone replacement therapy, operation history, hypertension, diabetes), and treatments (antihypertensive drugs, lipid treatment, insulin treatment).

### 2.3. CVD Outcomes Assessment

The aim of this study was to investigate the association between lifestyle and CVD risk in participants with breast cancer throughout the study period. CVD outcomes of interest included overall CVD, CHD, ischemic stroke (IS), and HF. Incident CHD and IS cases were ascertained using the 10th International Classification of Diseases Revision. The present study defined CHD and IS by codes I20–I25 and I63–I64, respectively. Furthermore, HF was defined by codes I11, I13, and I50. CVD outcomes were considered “terminated” for only participants diagnosed for the first time with any of these conditions during the UKB follow-up. Definitions and sources of detailed information for BC and CVD outcomes in UK Biobank are provided in Supplementary Table S1.

### 2.4. Definition of Genetic Risk Score

Bycroft et al. have summarized the detailed information about the genotyping, imputation, and quality control in UK Biobank [29]. In the present study, we generated the PRS to quantify the genetic risk of CHD, IS, and HF using single-nucleotide polymorphisms (SNPs) extracted from published genome-wide association studies, respectively. A weighted method was used to calculate the PRS and the β coefficients reported in previous studies for each SNP were used for weights [30]. The PRS for HF was built using 12 SNPs reported by Shah et al [31]. For CHD and IS, 64 SNPs [32] and 32 SNPs [33] previously reported were used to generate the PRS, respectively. Detailed information about the SNPs is listed in Supplementary Table S2.

### 2.5. Statistical Analysis

The baseline characteristics were presented according to the number of lifestyle factors. Continuous variables were described as mean and standard deviation. Categorical variables were described as absolute values and percentages. Missing data were coded as missing indicators for continuous variables and mean values for continuous variables. The duration of follow-up was defined as the time between the baseline and incident CVD event, or death or the censoring date (21 March 2021). For those who had more than one event during the study period, the first event date was used in the analysis. The Cox hazard regression model was performed to investigate the association between healthy lifestyle scores and CVD outcomes among females diagnosed with breast cancer. Multivariate-adjusted hazard ratio (HR) and 95% confidence interval (95%CI) for CVD outcomes were calculated using Cox proportional hazards regression models. In the primary analysis, models were adjusted for age at diagnosis of breast cancer (continuous), race (white European, others), the Townsend Deprivation Index (continuous), diabetes (yes/no), hypertension (yes/no), antihypertensive drugs (yes/no), insulin treatment (yes/no), lipid treatments (yes/no), hormone replacement therapy (HRT, yes/no), menopause (yes/no), and surgical treatment of breast cancer (yes/no). The Townsend Deprivation Index was regarded as an area-based proxy measure for socioeconomic status [34]. The linear trend test was performed by treating the healthy lifestyle score as a continuous variable.

In the analysis for PRS, we further adjusted for genetic testing batches and the first ten genetic principal components in the Cox proportional hazards model, to investigate the interaction of the gene–lifestyle score on CVD risk. The cross-product terms of the healthy lifestyle score with the CVD PRS were added in the models. Interactions were assessed using the likelihood ratio test. Subgroup analyses stratified by genetic risk were also performed.

Additionally, several sensitivity analyses were conducted to test the robustness of our findings. To estimate whether socioeconomic status might influence the results, we additionally adjusted for education (high school and below, graduate and above) and household income (<30,999, 31,000–10,000, >100,000, unknown) in the model. Furthermore, to avoid the reverse causality effect, we restricted the CVD cases to more than two years from baseline. A weighted standardized healthy lifestyle score was generated based on the β coefficients of each lifestyle factor with CVD outcomes. The weighted healthy lifestyle score was then used to perform sensitivity analysis.

All the analyses were performed using R 4.0.2. The statistical tests were two-sided, and the statistical significance threshold was set at 0.05 for *p* values.

## 3. Results

### 3.1. Participants’ Characteristics

The baseline characteristics of participants according to the number of healthy lifestyle factors are summarized in Table 1. A total of 13,348 females diagnosed with BC were included in the analysis, and the mean age at BC diagnosis was 52.4 years. Among the subjects, 95.7% were white Europeans, and their mean BMI was 26.9 (4.9). For the five lifestyle factors of interest, 39.3% did not have overweight/obesity, 91.9% were non-smokers, 71.0% drank moderately, 59.6% were physically active, and 50.2% had a healthy diet. Additionally, 4.8% (*n* = 640), 16.6% (*n* = 2217), 25.4% (*n* = 3391), and 29.9% (*n* = 3995) subjects had 0–1, 2, 3, and 4 or more healthy lifestyle factors.

During a median of 8.01 (interquartile range, 7.16–8.76) years of follow-up, 986 CVD events were identified, including 736 CHD, 165 IS, and 353 HF. The PRS of CHDs, IS, and HF approximated a normal distribution (Supplementary Figures S1–S3).

### 3.2. Healthy Lifestyle Score and the Risk of Incident CVD

**Figure 1:**
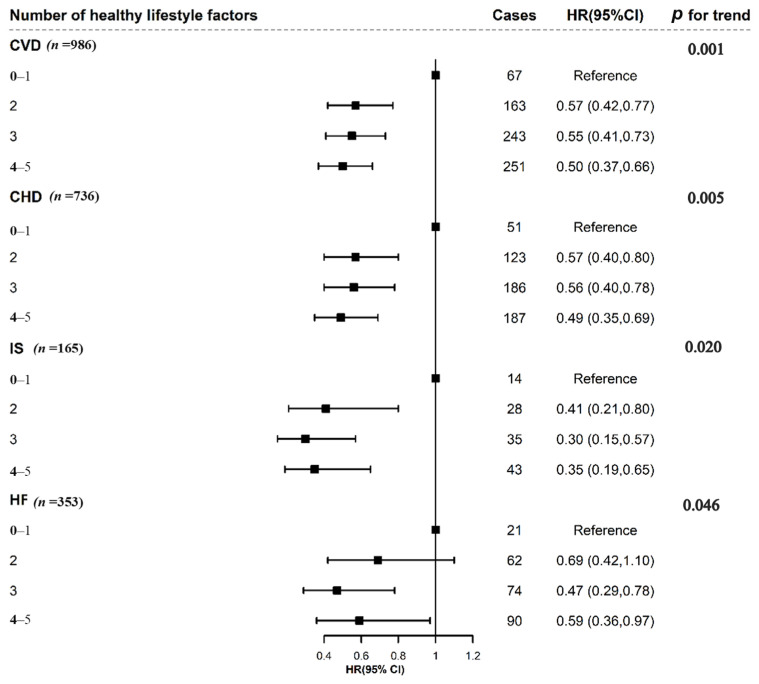
Multivariable adjusted hazard ratios for CVD event risks by number of healthy lifestyle factors. CVD, cardiovascular disease; CHD, coronary heart disease; IS, ischemic stroke; HF, heart failure; CI, confidence interval. The healthy lifestyle score was divided into four groups: 0–1, 2, 3, and 4–5. The model was adjusted for age at diagnosis of breast cancer, race (white European, others), the Townsend Deprivation Index (continuous), diabetes (yes/no), hypertension (yes/no), antihypertensive drugs (yes/no), insulin treatment (yes/no), lipid treatments (yes/no), HRT (yes/no), menopause (yes/no), surgical treatment of breast cancer (yes/no). *p* value for trend calculated treating the genetic risk score as a continuous variable.

In Figure 1, associations of the healthy lifestyle score with lower risk of CVD outcome are exhibited. As shown in Figure 1, a healthy lifestyle (4 or more favorable behaviors) was significantly associated with a lower risk of CVD (HR: 0.50; 95%CI: 0.37, 0.66) compared with an unhealthy lifestyle (0 or 1 favorable behaviors). Similarly, compared to participants with the lowest healthy lifestyle score, those with the highest healthy lifestyle score had a significantly lower risk of CHD (HR: 0.49; 95%CI: 0.35, 0.69), IS (HR: 0.35; 95%CI: 0.19, 0.65), and HF (HR: 0.59; 95%CI: 0.36, 0.97). Furthermore, when the healthy lifestyle score was treated as a continuous variable, the trend analysis also revealed that a higher healthy lifestyle score was significantly associated with a lower risk of CVD, CHD, IS, and HF (*p* trend < 0.05). Additionally, each single healthy lifestyle factor tended to be associated with a lower risk of CVD outcomes, and the results of individual lifestyle factors with CVD are presented in Supplementary Table S3.

### 3.3. Genetic Risk Score and Risk of Incident CVD

The PRS of different CVD outcomes were dichotomized into low risk and high risk according to the median PRS. The high genetic risk categories were associated with CHD (HR: 1.36, 95%CI: 1.10, 1.67), IS (HR: 1.25, 95%CI: 0.91, 1.72), and HF risk (HR: 1.40, 95%CI: 1.06, 1.84), with a higher risk score indicating high susceptibility to CVD outcomes.

### 3.4. Healthy Lifestyle Score, Genetic Risk Score, and Risk of Incident CVD

Then, we further investigated the interaction of genetic risk and lifestyle score on CVD outcomes. Statistically significant interactions were found between genetic risk and lifestyle score on CHD (*p* for interaction = 0.034) and HF (*p* for interaction = 0.044), respectively.

**Figure 2:**
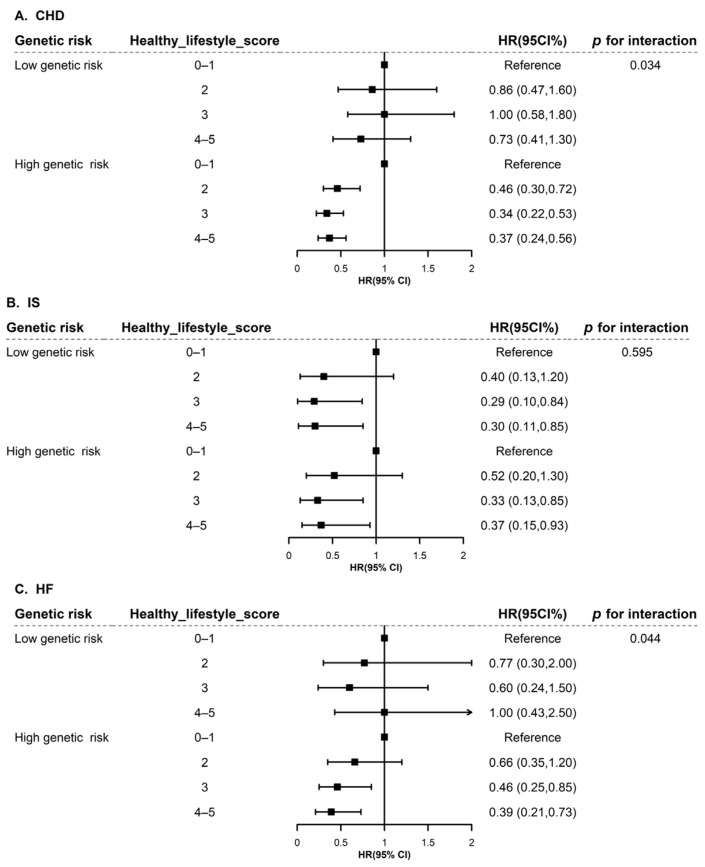
Subgroup analysis of healthy lifestyle score with CHD (**A**), IS (**B**), and HF (**C**) among females with breast cancer stratified by genetic risk. CHD, coronary heart disease; IS, ischemic stroke; HF, heart failure; CI, confidence interval. Genetic risk is classified as low risk or high risk based on median. We investigated the association of healthy lifestyle factors with CHD, IS, and HF stratified by genetic risk. The model was adjusted for age at diagnosis of breast cancer, race (white European, others), the Townsend Deprivation Index (continuous), diabetes (yes/no), hypertension (yes/no), antihypertensive drugs (yes/no), insulin treatment (yes/no), lipid treatments (yes/no), HRT (yes/no), menopause (yes/no), surgical treatment of breast cancer (yes/no), genotyping batch, and the first 10 genetic principal components.

Similarly, further results stratified by genetic risk category (low/high genetic risk) are presented in Figure 2. Compared with the reference group (0–1 healthy lifestyle score), it was suggested that having more healthy lifestyle factors was associated with a lower risk of incident CHD, IS, and HF (Figure 2). Notably, the results were more remarkable in the high genetic risk group. Among the participants with a high genetic risk, compared with those having a 0–1 healthy lifestyle score, the healthiest lifestyle score was significantly associated with a decreased risk of CHD (HR: 0.37; 95%CI: 0.24, 0.56), IS (HR: 0.37; 95%CI: 0.15, 0.93), and HF (HR: 0.39; 95%CI: 0.21, 0.73).

### 3.5. Sensitivity Analysis

We conducted several additional sensitivity analyses to evaluate the robustness of our findings and the results are presented in Supplementary Table S4. In the sensitivity analysis, the results remained robust after adjusting for household income and education: keeping a healthy lifestyle was associated with a lower risk of subsequent CVD among BC patients. The joint analysis showed that compared with participants having the least healthy lifestyles in the high genetic risk group, the participants with 3–5 healthy lifestyle scores and in the bottom 50% of genetic risk were significantly associated with a lower risk of CHD (HR: 0.66; 95%CI: 0.51, 0.87), IS (HR: 0.49; 95%CI: 0.27, 0.88), and HF (HR: 0.50; 95%CI: 0.34, 0.74) (Supplementary Table S4). Similarly, the results remained robust when the study excluded people who had developed the disease in the previous two years or used a weighted healthy lifestyle score (Supplementary Table S4). The above sensitivity analyses consistently observed significant interactions between genetics and lifestyle scores for HF (*p* < 0.05).

## 4. Discussion

Based on data from UK Biobank, healthy lifestyle factors were associated with a decreased risk of incident CVD among BC survivors. Notably, the cohort study firstly reported that the associations between a healthy lifestyle and incident CVD were stronger among BC survivors with a higher PRS. There were significant interactions between genetic risk and the lifestyle score in relation to CHD and HF.

The current study demonstrated that a healthy lifestyle was associated with a decreased risk of incident CVD among BC survivors. These associations have been documented previously among general populations, showing that adherence to a healthy lifestyle pattern could substantially lower the risk of CVD [35,36]. The associations were also validated in patients with diabetes mellitus [37] and hypertension [38]. The current study firstly reported consistent associations among BC survivors, where those with the healthiest lifestyle would have a 50% lower risk of incident CVD compared with the least healthy lifestyles. Multiple studies suggested that the adoption of combined healthy lifestyles benefits the prevention of incident CVD risk in both general and affected individuals [35,36,37,38]. BC survivors should follow a healthy lifestyle to avoid the burden of subsequent CVD onset and achieve a higher quality of survival.

The previous study has demonstrated that a healthy lifestyle could offset the genetic risk, and adherence to a healthy lifestyle might decrease by 50% the risk of CHD compared with an unfavorable lifestyle among participants at high genetic risk [23]. Similarly, a UK Biobank study suggested that an unhealthy lifestyle might increase the risk of CVD [39]. Our study provided consistent findings among BC survivors, largely in line with the general population [7,22,40]. The lifestyle–genetic interaction was not observed in previous studies [22,41,42,43], while we found a significant lifestyle–genetic interaction for CHD, suggesting that people at a higher genetic risk of CVD could benefit more from lifestyle adherence.

Similar lifestyle–genetic interaction patterns were also observed for HF in the current study. A previous study conducted in BC survivors from the Women’s Health Initiative summarized that the risk factors for HF were largely similar to those in the entire population [13]. However, they did not focus on the combination of modifiable lifestyle factors and the potential interaction of lifestyle and genetic risk [13]. The Women’s Health Initiative’s findings supported that a healthy lifestyle was associated with decreased HF risk among postmenopausal women [44]. A UK Biobank study reported that combined healthy lifestyle factors were associated with a lower risk of incident HF across different genetic risk groups [45], which was consistent with the current study. However, there was limited evidence for a lifestyle–genetic interaction for HF. Our findings provided new insights that BC survivors with a higher genetic risk could benefit more from lifestyle modifications in reducing the risk of incident CHD and HF.

Leveraging the UK Biobank study, Loes reported that the entire population with a high genetic risk combined with an unhealthy lifestyle might carry a more than twofold increased risk of stroke compared to those with a low genetic risk combined with a healthy lifestyle [40]. However, there was limited evidence for an interaction between PRS and lifestyle score for IS, which was in line with our findings. Two previous UK Biobank studies suggested that a lifestyle–gene interaction was found for stroke [39,46]. However, they did not focus on IS, and the relatively small number of cases of IS might limit the power of the analysis. Nonetheless, the existing evidence suggests that a reduction in CVD risk by adherence to a healthy lifestyle pattern can be achieved in the general population or those who suffer from BC conditions, regardless of genetic risk.

Several mechanisms have been proposed to explain lifestyles, genetic factors, and CVD risk. A review summarized that lifestyle might play a more dominant role in the development of CVD, though the impact of genetic factors is also critical [47]. Twin and familial aggregation studies have confirmed that CVD is heritable, while genome-wide association studies have investigated the potential genetic variants associated with CVD [48,49,50]. CVD risk from genetic factors is often difficult to change. However, a substantial part of the risk (50–90%) of CVD events could be prevented by maintaining a healthy lifestyle [47,51,52]. Inflammation and oxidative stress were summarized as the most essential mechanisms in the development of CVD, and clinical trials suggest that this association is causal [53,54]. Anti-inflammatory milieu and anti-atherogenic effects in the vasculature, as well as oxidative stress, explain most of the reasons that a healthy lifestyle prevents CVD. In addition, a healthy lifestyle pattern might contribute to the development of CVD through other mechanisms, including the gut microbiome, which modulates host inflammation and metabolism, oxidative damage, and endothelial dysfunction [55,56,57,58,59].

To our knowledge, this is the first study to demonstrate that BC survivors, as a high-risk population with regard to CVD, should adhere to a healthy lifestyle. The primary strength of the study was the creation of the PRS to investigate the interaction between genetic risk and healthy lifestyle scores among BC patients. Our study provides new evidence to help prevent high CVD risk among patients with BC and suggests that keeping a healthy lifestyle might help to offset the genetic risk of CVD in cancer patients. However, we also acknowledge several limitations. First of all, the lifestyle score has not been validated except in the current study. However, previous studies have generally used the same approach to calculate healthy lifestyle scores [22,26,41]. Secondly, the participants included were mainly white Europeans, which might limit the generalizability of our findings. Thirdly, potential confounders were included as covariates, but unmeasured and residual confounding factors cannot be avoided. Additionally, self-reported information on lifestyle might lead to misclassification errors. Fourthly, the study population was limited to BC survivors, and the incidence of CVD events was relatively low, such as with IS. Future studies are needed to validate our findings. Lastly, BC treatment may increase the risk of cardiac dysfunction in patients. Due to the lack of treatment-related data, we only adjusted for surgery and estrogen replacement therapy in the models [60].

## 5. Conclusions

Adherence to a healthy lifestyle is associated with a decreased risk of subsequent CVD and might offset the genetic risk of CVD among females with BC. Our findings highlight the urgent need for healthy lifestyle management among individuals with BC for CVD prevention. Further studies should focus on the role of longitudinal lifestyle changes in CVD morbidity and mortality in individuals with BC.

## Figures and Tables

**Table 1:** Baseline characteristics of the study participants according to number of healthy lifestyle factors.

Characteristics	Overall	Number of Healthy Lifestyle Factors				*p*
		0–1	2	3	4–5	
Number of participants (%)	13,348 (100.0)	640 (4.8)	2217 (16.6)	3391 (25.4)	3995 (29.9)	
Age (Years, mean (SD))	58.80 (7.1)	56.63 (7.2)	58.26 (7.1)	58.57 (7.2)	58.81 (7.2)	<0.001
BMI (kg/m^2^, mean (SD))	26.9 (4.9)	29.3 (4.5)	28.8 (5.0)	27.3 (4.7)	24.7 (3.9)	<0.001
Age diagnosed with BC (Years, mean (SD))	52.4 (8.6)	51.1 (8.4)	52.2 (8.6)	52.2 (8.6)	52.1 (8.6)	0.056
White European (%)	12780 (95.7)	629 (98.3)	2156 (97.2)	3241 (95.6)	3812 (95.4)	<0.001
Income (%)						0.04
<30,999	6051 (45.3)	294 (45.9)	1047 (47.2)	1624 (47.9)	1772 (44.4)	
31,000–10,000	4323 (32.4)	243 (38.0)	773 (34.9)	1178 (34.7)	1440 (36.0)	
>100,000	413 (3.1)	23 (3.6)	66 (3.0)	112 (3.3)	157 (3.9)	
unknown	2561 (19.2)	80 (12.5)	331 (14.9)	477 (14.1)	626 (15.7)	
Education years (>15 years, %)	7427 (56.7)	319 (50.2)	1202 (54.7)	1980 (58.8)	2553 (64.5)	<0.001
BMI < 25(%)	5243 (39.3)	42 (6.6)	350 (15.8)	1085 (32.0)	2713 (67.9)	<0.001
Non-current smoker (%)	12261 (91.9)	359 (56.1)	1898 (85.6)	3212 (94.7)	3948 (98.8)	<0.001
Non-excessive alcohol intake (%)	9483 (71.0)	97 (15.2)	1198 (54.0)	2378 (70.1)	3525 (88.2)	<0.001
Being physically active (%)	6107 (59.6)	53 (8.3)	610 (27.5)	1931 (56.9)	3513 (87.9)	<0.001
Healthy dietary habits (%)	6700 (50.2)	25 (3.9)	378 (17.1)	1567 (46.2)	3335 (83.5)	<0.001
Dietary metrics, %						
Vegetables ≥ 4 servings/day	9412 (71.7)	348 (54.8)	1316 (59.8)	2379 (70.6)	3353 (84.6)	<0.001
Fruits ≥ 3 servings/day	7957 (60.4)	185 (29.0)	929 (42.1)	1989 (59.0)	3214 (80.9)	<0.001
Fish ≥ 2 servings/week	7540 (57.0)	236 (37.0)	922 (41.9)	1845 (54.6)	2873 (72.1)	<0.001
Red meat ≤ 2 servings/week	9305 (70.4)	321 (50.3)	1264 (57.6)	2345 (69.4)	3302 (82.9)	<0.001
Processed meat ≤ 1 serving/week	10702 (80.4)	436 (68.1)	1541 (69.6)	2701 (79.8)	3630 (90.9)	<0.001
Menopause (%)	1214 (9.1)	76 (11.9)	206 (9.3)	329 (9.7)	383 (9.6)	0.001
HRT (%)	8116 (60.8)	407 (63.6)	1351 (60.9)	2068 (61.0)	2499 (62.6)	0.137
Hypertension history (%)	3291 (24.7)	150 (23.4)	623 (28.1)	840 (24.8)	766 (19.2)	<0.001
Diabetes history (%)	538 (4.0)	21 (3.3)	123 (5.5)	132 (3.9)	102 (2.6)	<0.001
Treatment/medication (%)						
Antihypertensive drugs	3291 (24.7)	150 (23.4)	623 (28.1)	840 (24.8)	766 (19.2)	<0.001
Insulin treatment	538 (4.0)	21 (3.3)	123 (5.5)	132 (3.9)	102 (2.6)	<0.001
Lipid treatment	1593 (11.9)	73 (11.4)	289 (13.0)	411 (12.1)	366 (9.2)	<0.001
Operative treatment (%)	9515 (71.3)	478 (74.7)	1632 (73.6)	2411 (71.1)	2793 (69.9)	0.004

SD, standard deviation; BMI, body mass index; BC, breast cancer; HRT, hormone replacement therapy. For alcohol drinking, those with a daily alcohol consumption <14 g were defined as non-excessive alcohol drinkers. Being physically active included those engaged in at least 150 min of moderate physical activity or at least 75 min of vigorous physical activity per week.

## Data Availability

Data for this study are available by request from the UK Biobank website (https://biobank.ctsu.ox.ac.uk/ (accessed on 20 December 2022)).

## Supplementary Materials

The following supporting information can be downloaded at: https://www.mdpi.com/article/10.3390/nu15040864/s1, Figure S1: Distribution of the polygenic risk score for coronary heart disease; Figure S2: Distribution of the polygenic risk score for ischemic stroke; Figure S3: Distribution of the polygenic risk score for heart failure. Table S1: Codes used in the UK Biobank study to identify breast cancer and CVD cases; Table S2: Detail information of SNP for genetic risk score; Table S3: Association between individual lifestyle factors and the risk of incident CVD; Table S4: The joint association of genetic risk and healthy lifestyle factors with CHD, IS and HF among females with breast cancer in three sensitivity analysis.
