# Supplementary material for: Lifestyle Factors, Genetic Risk, and Cardiovascular Disease Risk among Breast Cancer Survivors: A Prospective Cohort Study in UK Biobank
Source: Nutrients. 2023 Feb 8;15(4):864. doi: 10.3390/nu15040864 (PMC9965301; doi:10.3390/nu15040864)
Supplement: Supplementary file 1 [file nutrients-15-00864-s001.zip › nutrients-2153863-supplementary.pdf]

Supplementary materials

Supplementary Tables S1–S4; Supplementary Figures S1–S3.

Supplementary Table S1 Codes used in the UK Biobank study to identify breast cancer and CVD cases

Cases	ICD-9	ICD-10	Self-reported UK Biobank field code
BC	174, 2330	C50	1002
CVD	410,411,412,414,434,435,431,430	I20-I25, I60-I64, G45	1081,1583,1082,1491,1086
CHD	410,411,412,414	I20-I25	1070,1074,1075,1095,1523
IS	434	I63, I64	6150
HF	428	I11, I13, I50	1076

BC, breast cancer; CHD, coronary heart disease; IS, ischemic stroke; HF, heart failure; International Classification of Diseases, 10th edition (ICD-10); International Classification of Diseases, 9th edition (ICD-9).

Supplementary Table S2 Detail information of SNP for genetic risk score

Outcomes	SNP	CHR	EA	β
CHD	rs11206510	1	T	0.0770
CHD	rs17114036	1	A	0.1222
CHD	rs646776	1	T	0.1044
CHD	rs4845625	1	T	0.0488
CHD	rs17464857	1	T	0.0583
CHD	rs17465637	1	C	0.0770
CHD	rs16986953	2	A	0.0862
CHD	rs515135	2	C	0.0677
CHD	rs6544713	2	T	0.0488
CHD	rs1561198	2	T	0.0583
CHD	rs2252641	2	C	0.0296
CHD	rs6725887	2	C	0.1310
CHD	rs9818870	3	T	0.0677
CHD	rs1878406	4	T	0.0583
CHD	rs7692387	4	G	0.0677
CHD	rs273909	5	G	0.0583
CHD	rs6903956	6	A	0.0000
CHD	rs12526453	6	C	0.0953
CHD	rs17609940	6	G	0.0296
CHD	rs10947789	6	T	0.0488
CHD	rs12190287	6	C	0.0583
CHD	rs2048327	6	C	0.0583
CHD	rs4252120	6	T	0.0296
CHD	rs2023938	7	C	0.0583
CHD	rs10953541	7	C	0.0488
CHD	rs11556924	7	C	0.0770
CHD	rs264	8	G	0.0583
CHD	rs2954029	8	A	0.0392
CHD	rs3217992	9	T	0.1310
CHD	rs4977574	9	G	0.1906
CHD	rs579459	9	C	0.0770
CHD	rs2505083	10	C	0.0583
CHD	rs2047009	10	G	0.0583
CHD	rs501120	10	T	0.0770
CHD	rs11203042	10	T	0.0392
CHD	rs1412444	10	T	0.0677
CHD	rs12413409	10	G	0.0770
CHD	rs974819	11	T	0.0677
CHD	rs964184	11	G	0.0488
CHD	rs7136259	12	T	0.0392

Outcomes	SNP	CHR	EA	β
CHD	rs3184504	12	T	0.0677
CHD	rs9319428	13	A	0.0392
CHD	rs4773144	13	G	0.0488
CHD	rs9515203	13	T	0.0677
CHD	rs2895811	14	C	0.0392
CHD	rs7173743	15	T	0.0770
CHD	rs17514846	15	A	0.0488
CHD	rs216172	17	C	0.0488
CHD	rs12936587	17	G	0.0296
CHD	rs46522	17	T	0.0392
CHD	rs1122608	19	G	0.0770
CHD	rs2075650	19	G	0.0677
CHD	rs445925	19	G	0.0862
CHD	rs9982601	21	T	0.1133
CHD	rs17087335	4	T	0.0583
CHD	rs3918226	7	T	0.1310
CHD	rs10840293	11	A	0.0583
CHD	rs56062135	15	C	0.0677
CHD	rs8042271	15	G	0.0953
CHD	rs7212798	17	C	0.0770
CHD	rs663129	18	A	0.0583
CHD	rs180803	22	G	0.1823
CHD	rs11830157	12	G	0.0392
CHD	rs12976411	19	T	-0.0513
IS	rs880315	1	C	0.0488
IS	rs12037987	1	C	0.0677
IS	rs146390073	1	T	0.6678
IS	rs12476527	2	G	0.0488
IS	rs7610618	3	T	0.8459
IS	rs34311906	4	C	0.0677
IS	rs17612742	4	C	0.1740
IS	rs6825454	4	C	0.0583
IS	rs11957829	5	A	0.0677
IS	rs6891174	5	A	0.1044
IS	rs16896398	6	T	0.0488
IS	rs42039	7	C	0.0677
IS	rs7859727	9	T	0.0488
IS	rs10820405	9	G	0.1823
IS	rs2295786	10	A	0.0488
IS	rs7304841	12	A	0.0488
IS	rs35436	12	C	0.0488

Outcomes	SNP	CHR	EA	β
IS	rs9526212	13	G	0.0583
IS	rs4932370	15	A	0.0488
IS	rs11867415	17	G	0.0862
IS	rs2229383	19	T	0.0488
IS	rs8103309	19	T	0.0488
IS	rs12124533	1	T	0.1570
IS	rs1052053	1	G	0.0583
IS	rs13143308	4	T	0.2776
IS	rs4959130	6	A	0.0770
IS	rs2107595	7	A	0.1906
IS	rs635634	9	T	0.0770
IS	rs2005108	11	T	0.0770
IS	rs3184504	12	T	0.0770
IS	rs12932445	16	C	0.1823
IS	rs12445022	16	A	0.0583
HF	rs11745324	5	G	0.0488
HF	rs140570886	6	C	0.2151
HF	rs1556516	9	C	0.0583
HF	rs17042102	4	A	0.1133
HF	rs17617337	10	C	0.0583
HF	rs4135240	6	T	0.0488
HF	rs4746140	10	G	0.0677
HF	rs4766578	12	T	0.0392
HF	rs55730499	6	T	0.1044
HF	rs56094641	16	G	0.0488
HF	rs600038	9	C	0.0583
HF	rs660240	1	C	0.0583

CHD, coronary heart disease; IS, ischemic stroke; HF, heart failure; SNP, single nucleotide polymorphism; CHR, chromosome; EA, effect allele

Supplementary Table S3 Association between individual lifestyle factors and the risk of incident CVD

Lifestyle factors	Outcomes			
	CVD	CHD	IS	HF
BMI>=25kg/m^2	Reference	Reference	Reference	Reference
**BMI<25kg/m^2**	0.79 (0.66,0.96)	0.73 (0.59,0.90)	0.88 (0.55,1.40)	0.74 (0.55,1.00)
Current smoking	Reference	Reference	Reference	Reference
**Non-current smoking**	0.70 (0.59,0.83)	0.73 (0.60,0.89)	0.85 (0.56,1.30)	0.90 (0.68,1.20)
Excessive alcohol intake	Reference	Reference	Reference	Reference
**Non-excessive alcohol intake**	1.00 (0.83,1.20)	1.00 (0.81,1.20)	0.81 (0.52,1.20)	0.95 (0.70,1.30)
Physical inactive	Reference	Reference	Reference	Reference
**Physical active**	0.90 (0.76,1.10)	0.89 (0.74,1.10)	0.85 (0.57,1.30)	0.91 (0.69,1.20)
Unhealthy dietary habit	Reference	Reference	Reference	Reference
**Healthy dietary habit**	0.92 (0.76,1.10)	0.91 (0.72,1.10)	0.81 (0.50,1.30)	0.71 (0.52,0.96)

Models were adjusted for age at diagnosis of breast cancer (continuous), race (white European, others), the Townsend Deprivation Index (continuous), education years (≤15years, >15years), income (<30999,31000-10000, >100000, unknown), diabetes (yes/no), hypertension (yes/no), antihypertensive drugs (yes/no), insulin treatment (yes/no), lipid treatments (yes/no), lipid treatments (yes/no), hormone replacement therapy (HRT, yes/no), menopause(yes/no), surgical treatment of breast cancer (yes/no).

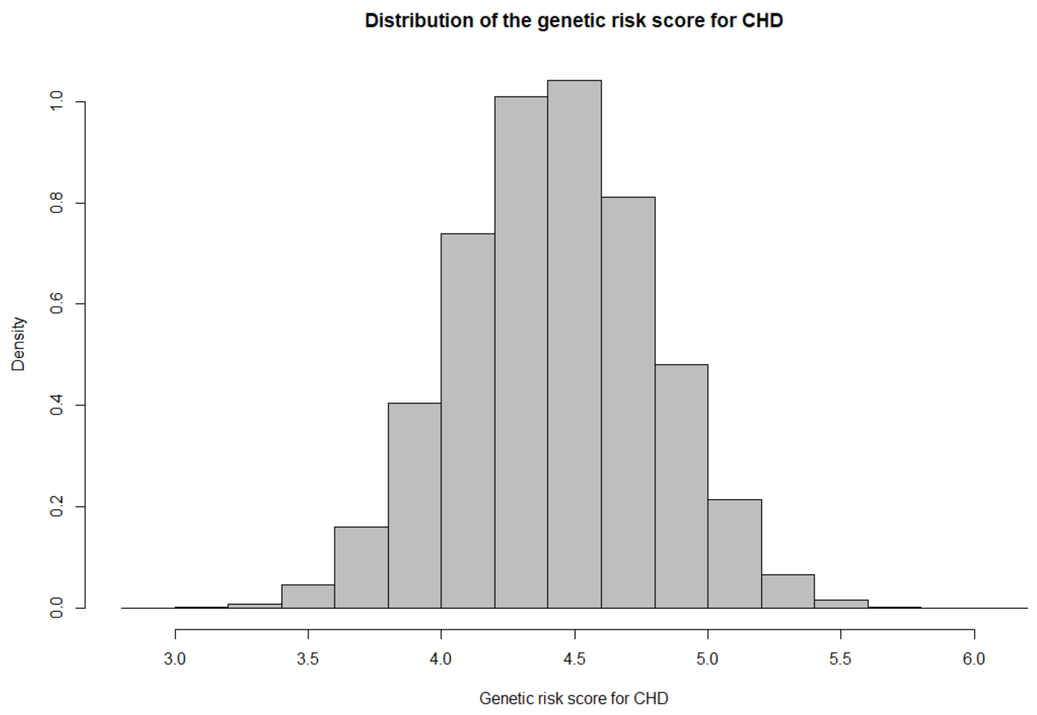

Supplementary Figure S1. Distribution of the polygenic risk score for coronary heart disease.

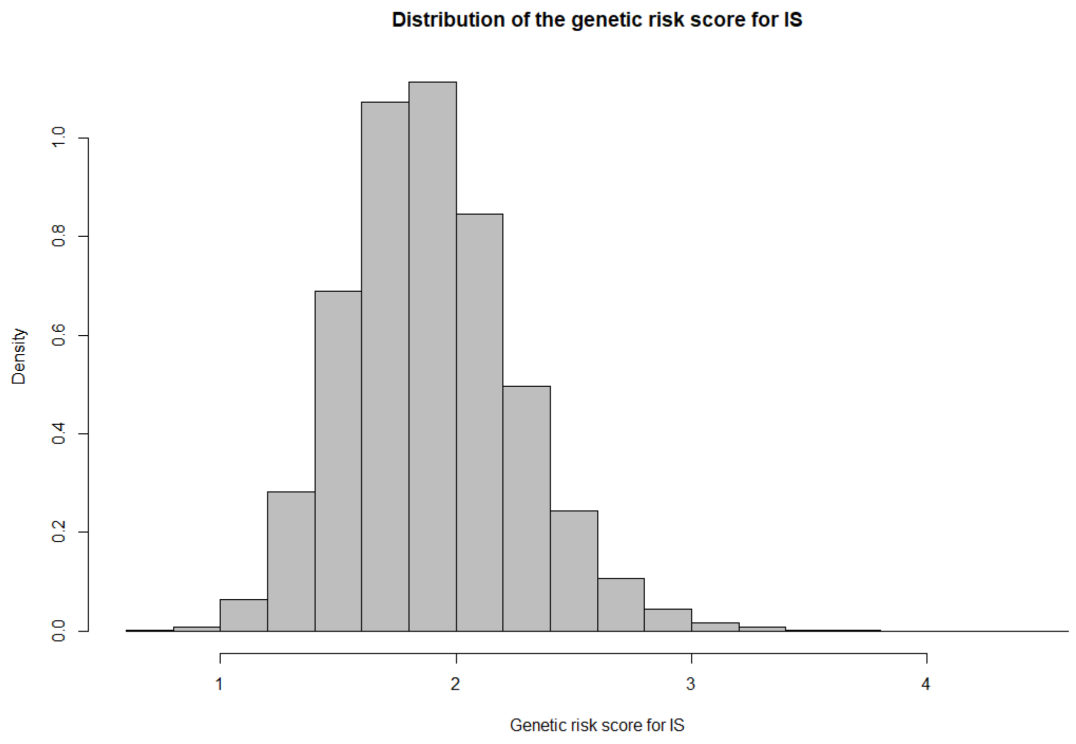

Supplementary Figure S2. Distribution of the polygenic risk score for ischemic stroke.

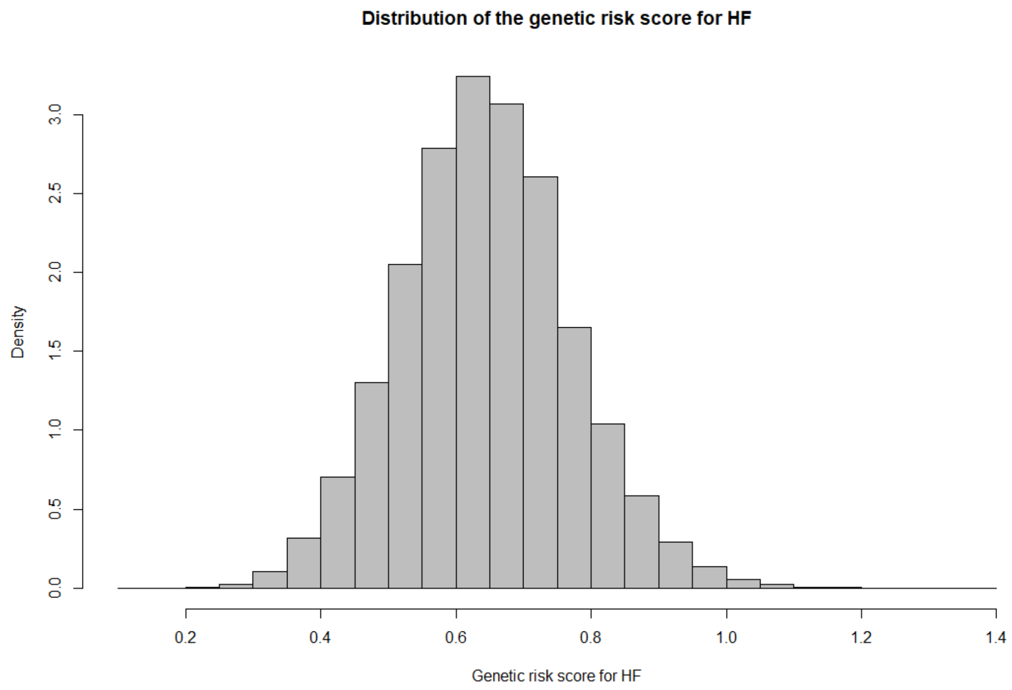

Supplementary Figure S3. Distribution of the polygenic risk score for heart failure

Supplementary Table S4 The joint association of genetic risk and healthy lifestyle factors with CHD, IS and HF among females with breast cancer in three sensitivity analysis

Outcomes	Group	N/cases	Primary analysis		Sensitivity analysis 1		Sensitivity analysis 2		Sensitivity analysis 3	
			HR (95%CI)	P_{inter}	HR (95%CI)	P_{inter}	HR (95%CI)	P_{inter}	HR (95%CI)	P_{inter}
**A. CHD**				0.034		0.032		0.101		0.101
	**High genetic risk**									
	0-2	1371/98	Reference		Reference		Reference		Reference	
	3-5	1345/71	0.66 (0.50,0.87)		0.66 (0.50,0.87)		0.70 (0.52,0.94)		0.66 (0.50,0.87)	
	**Low genetic risk**									
	0-2	3493/181	0.69 (0.50,0.97)		0.69 (0.50,0.97)		0.77 (0.54,1.10)		0.69 (0.50,0.97)	
	3-5	3509/168	0.66 (0.51,0.87)		0.66 (0.51,0.87)		0.73 (0.54,0.98)		0.66 (0.51,0.87)	
**B. IS**				0.595		0.520		0.174		0.174
	**High genetic risk**									
	0-2	1410/22	Reference		Reference		Reference		Reference	
	3-5	1306/18	0.51 (0.28,0.91)		0.51 (0.28,0.91)		0.50 (0.27,0.94)		0.51 (0.28,0.91)	
	**Low genetic risk**									
	0-2	3467/41	0.81 (0.42,1.60)		0.81 (0.42,1.60)		0.84 (0.42,1.70)		0.81 (0.42,1.60)	
	3-5	3535/32	0.49 (0.27,0.88)		0.49 (0.27,0.88)		0.49 (0.26,0.91)		0.49 (0.27,0.88)	
**C. HF**				0.044		0.037		0.021		0.038
	**High genetic risk**									
	0-2	1340/50	Reference		Reference		Reference		Reference	
	3-5	1376/28	0.59 (0.41,0.85)		0.59 (0.41,0.85)		0.56 (0.39,0.81)		0.59 (0.41,0.85)	
	**Low genetic risk**									
	0-2	3535/89	0.48 (0.30,0.78)		0.48 (0.3,0.78)		0.42 (0.25,0.70)		0.48 (0.30,0.78)	
		3467/69	0.50 (0.34,0.74)		0.50 (0.34,0.74)		0.47 (0.32,0.70)		0.50(0.34,0.74)	

Note: CHD, coronary heart disease; IS, ischemic stroke; HF, heart failure; CI, confidence interval. The healthy lifestyle score was divided into two groups: 0-2 and 3-5. Therefore, the final combined group was divided into 4 groups, with those with a score ≤2 and a genetic risk in the top 50% as the reference group.

The model was adjusted for adjusted for age at diagnosis of breast cancer (continuous), race (white European, others), the Townsend Deprivation Index (continuous), diabetes (yes/no), hypertension (yes/no), antihypertensive drugs (yes/no), insulin treatment (yes/no), lipid treatments (yes/no), hormone replacement therapy (HRT, yes/no), menopause(yes/no), surgical treatment of breast cancer (yes/no)., genotyping batch, and the first 10 genetic principal components.

The sensitivity analysis 1 was additionally adjusted for education years (≤15years, >15years), income (<30999,31000-10000, >100000, unknown) based on the primary analysis. The sensitivity analysis 2 was conducted when the participants with incident CVD in the previous 2 years were additionally excluded based on the sensitivity analysis 1. The sensitivity analysis 3 was performed using the weighted lifestyle factors score on the sensitivity analysis 1.
